# Aurora Kinase Inhibition Overcomes Cetuximab Resistance in Squamous Cell Cancer of the Head and Neck

**DOI:** 10.18632/oncotarget.311

**Published:** 2011-08-23

**Authors:** Alexander Hoellein, Anja Pickhard, Fabienne von Keitz, Stephanie Schoeffmann, Guido Piontek, Martina Rudelius, Anja Baumgart, Stefan Wagenpfeil, Christian Peschel, Tobias Dechow, Henning Bier, Ulrich Keller

**Affiliations:** ^1^III. Medical Department, Technische Universität München, Munich, Germany; ^2^Department of Head and Neck Surgery, Technische Universität München, Munich, Germany; ^3^Institute of Pathology, Technische Universität München, Munich, Germany; ^4^Institute for Medical Statistics and Epidemiology, Technische Universität München, Munich, Germany

**Keywords:** Squamous cell cancer of the head and neck, Aurora kinase, EGFR

## Abstract

Squamous cell cancer of the head and neck (SCCHN) is the sixth leading cause for cancer deaths worldwide. Despite extense knowledge of risk factors and pathogenesis about 50 percent of all patients and essentially every patient with metastatic SCCHN eventually die from this disease. We analyzed the clinical data and performed immunohistochemistry for Epidermal growth factor receptor (EGFR) and Aurora kinase A (Aurora-A) expression in 180 SCCHN patients. Patients characterized by elevated EGFR and elevated Aurora-A protein expression in tumor tissue represent a risk group with poor disease-free and overall survival (EGFR^low^ Aurora-A^low^ versus EGFR^high^ Aurora-A^high^, p = 0.024). Treating SCCHN cell lines with a pan-Aurora kinase inhibitor resulted in defective cytokinesis, polyploidy and apoptosis, which was effective irrespective of the EGFR status. Combined Aurora kinase and EGFR targeting using a monoclonal anti-EGFR antibody was more effective compared to single EGFR and Aurora kinase inhibition. Comparing pan-Aurora kinase and Aurora-A targeting hints towards a strong and clinically relevant biological effect mediated via Aurora kinase B. Taken together, our findings characterize a new poor risk group in SCCHN patients defined by elevated EGFR and Aurora-A protein expression. Our results demonstrate that combined targeting of EGFR and Aurora kinases represents a therapeutic means to activate cell cycle checkpoints and apoptosis in SCCHN.

## INTRODUCTION

Squamous cell cancer of the head and neck (SCCHN) is the sixth leading cause for cancer deaths worldwide [1]. Despite recent progress in understanding SCCHN biology and improved treatment, the 5 year survival has remained 50 percent for the past two decades. There is a pressing need to improve therapy in particular for patients with metastatic disease or local recurrence, where the median progression-free and overall survival is only ~ 6 months and ~11 months, respectively [2-4].

Several genetic alterations have been described in SCCHN, including mutations in the p53 tumor suppressor gene and mutations in genes that encode cell cycle proteins such as p16 and cyclin D1. In addition, several oncogenic pathways including Ras, PI3K/PTEN/Akt, TGF-β/BMP and EGFR/STAT3 are up-regulated in SCCHN [4-11]. Epidermal growth factor receptor (EGFR) overexpression in SCCHN is often caused by gene amplification [12], and elevated expression correlates with poor disease control and metastasis [13-14]. Furthermore, overexpression of two of its ligands, EGF and transforming growth factor-alpha (TGF-alpha), has been linked to a poor prognosis [15]. The major signaling pathways activated by EGFR are the RAS-RAF-MAP kinase pathway, which is mainly involved in proliferation, and the PI3K-PTEN-AKT pathway, which is mainly involved in survival [16]. The addition of the monoclonal antibody C225 (cetuximab) to the standard first-line regimen cisplatin/5-fluorouracil [17] not only increased the rate of objective responses but also improved progression-free and overall survival in patients with recurrent or metastatic SCCHN [2].

The Aurora kinases A and B (Aurora-A and Aurora-B) are highly conserved serine/threonine kinases that play essential and distinct roles in mitosis [18]. Specifically, Aurora-A is required for the assembly of the mitotic spindle, where it accumulates on centrosomes at the spindle poles during prophase until metaphase. Recently a kinase-independent role in mitotic spindle assembly has been reported for Aurora-A [19]. Aurora-B is required for mitotic progression and cytokinesis, and is localized, along with inner centromeric protein (INCENP) and survivin, at centromeres and the spindle midzone during the metaphase to anaphase transition [18, 20]. *AURORA-A (AURKA)* mRNA is amplified in a variety of human cancers including SCCHN, where it is associated with poor prognosis [21]. Increased levels of Aurora-B have been reported in various aggressive malignancies [20].

Both Aurora-A and EGFR overexpression have been implicated in SCCHN tumorigenesis and are established adverse prognostic factors. Aurora-A and EGFR share downstream signaling pathways, and each by itself represents an attractive therapeutic target. Here we report that joint protein overexpression of EGFR and Aurora-A defines a poor risk group among SCCHN patients. Combining drugs that target Aurora kinases and EGFR may overcome resistance to single agent treatment in SCCHN cells.

## RESULTS

### High levels of EGFR and Aurora-A assessed by IHC identify adverse prognosis in SCCHN

**Figure 1:**
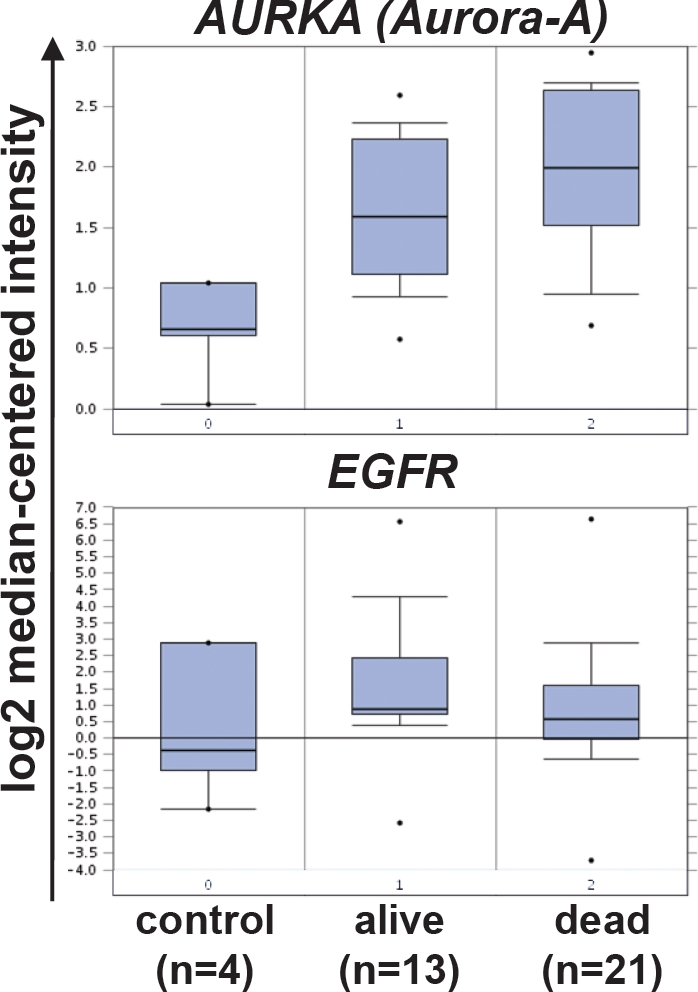
*EGFR* and *Aurora-A* transcript levels in SCCHN and clinical outcome A public database (www.oncomine.com) was searched for gene expression analyses studies that compare *AURORA-A* transcript levels in control tissue and SCCHN samples from patients who were alive or dead [22]. Shown is the log2 median-centered relative intensity of expression for *AURORA-A* (*AURKA*, upper panel, tumor versus control tissue: p = 0.002, [reporter: 34851_at]) and *EGFR* (lower panel, tumor versus control tissue: n.s., [reporter: 1537_at]).

**Figure 2:**
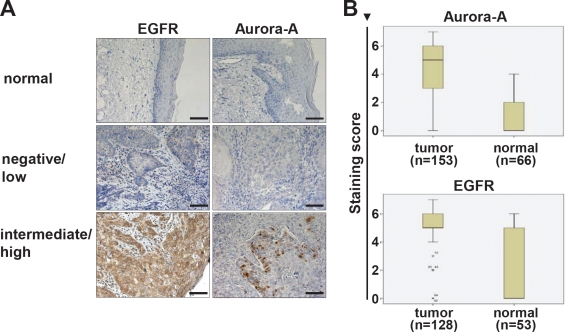
EGFR and Aurora-A expression in tumor tissue and adjacent normal mucosa (A) Histological assessment of EGFR and Aurora-A protein expression by immunohistochemistry. Shown are representative tumor samples that were graded as negative/low expression (middle panel), high expression (lower panel) and normal mucosa control tissue (upper panel). Bar equals 100 μm. (B) Within each patient sample the expression of Aurora-A and EGFR was assessed in normal adjacent tissue and tumor tissue. The differences are highly significant. Aurora-A: p<0.001; EGFR: p<0.001. The staining score is defined in the material and method section.

**Figure 3:**
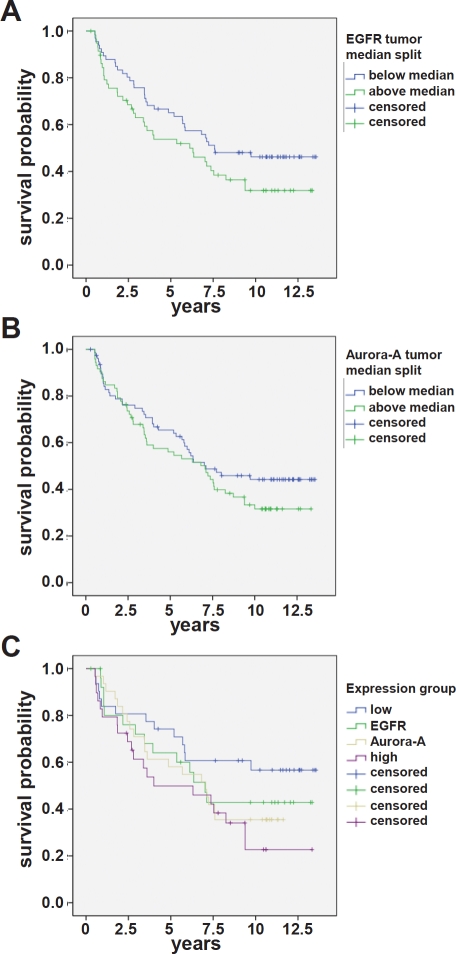
EGFR and Aurora-A expression assessed by IHC is an adverse prognostic factor in SCCHN (A) EGFR: the difference in disease-free survival for patients with expression above median (green curve; n = 90) is not statistically different from the survival of patients with expression below median (blue curve; n = 90). p = 0.10. (B) Aurora-A: the difference in disease-free survival for patients with expression below median (blue curve; n = 90) is not statistically different from the survival of patients with expression above median (green curve; n = 90). p = 0.21. (C) The difference in disease-free survival of patients with EGFR^high^ and Aurora-A^high^ is statistically different from the survival of patients who are characterized by EGFR^low^ and Aurora-A^low^. p = 0.024. The staining score is defined in the material and method section.

Publicly available gene expression data [22] (www.oncomine.org) were analyzed for the expression and prognostic relevance of *EGFR* and *AURORA-A* expression.* AURORA-A* transcripts were expressed at significantly higher levels in SCCHN tumor samples as compared to normal control tissue (p = 0.002, Figure 1), and the median relative expression in surviving patients was lower as compared to patients dying from SCCHN (n.s.). In a previous report the level of *AURORA-A*transcript was associated with survival [21]. We therefore next addressed the prognostic relevance of Aurora-A and EGFR protein levels in the SCCHN patient cohort (n=180) described in Table 1. There was a highly significant difference between patients' protein levels when comparing normal adjacent mucosa with the levels expressed in tumor cells for both Aurora-A and EGFR (Figure 2A), with independent expression of EGFR and Aurora-A for each patient (r = 0.03 / p = 0.74). Furthermore, there were clear differences in expression levels for Aurora-A and EGFR within the patient tumor tissue assessed (Figure 2). While protein levels of EGFR (Figure 3A) or Aurora-A (Figure 3B) above median assessed by IHC in a Kaplan Meier analysis did not identify a population with a significantly reduced disease-free survival (EGFR: p = 0.10; Aurora-A; p = 0.21), our analysis identifies a poor risk population with regard to overall and disease-free survival that is characterized by above median levels of EGFR (EGFR^high^) and Aurora-A (Aurora-A^high^) (p = 0.024, compared to EGFR^low^ and Aurora-A^low^, Figure 2C). Thus, the coexpression of elevated levels of Aurora-A and EGFR is an adverse prognostic factor in SCCHN.

**Table 1:** Patient characteristics (n = 180)

characteristic		number (%)
sex	female	17 (9)
	male	163 (91)
localisation	oral cavity	33 (18)
	oropharynx	58 (32)
	hypopharynx	33 (18)
	larynx	56 (31)
primary tumor category (pT)	pT1	25 (14)
	pT2	66 (37)
	pT3	48 (27)
	pT4	41 (23)
lymph node category (c/pN)	c/pN0	94 (52)
	pN1	23 (13)
	pN2a	2 (1)
	pN2b	39 (22)
	pN2c	20 (11)
	pN3	2 (1)
tumor grade	G1	10 (6)
	G2	110 (61)
	G3	60 (33)

### Aurora kinase inhibition results in defective cytokinesis and polyploidy irrespective of the EGFR status

**Figure 4:**
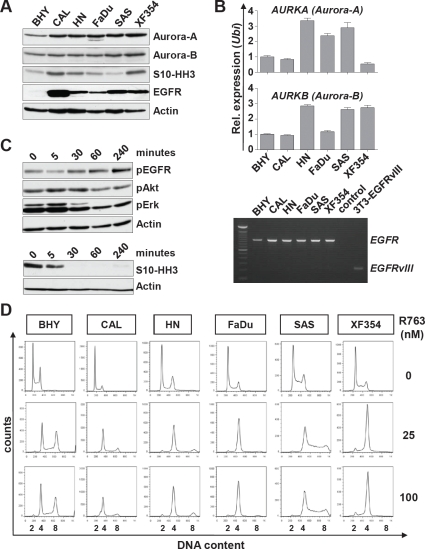
Expression and activity of Aurora kinases and EGFR in SCCHN cell lines (A) Six SCCHN cell lines were assessed by immunoblotting for the expression of Aurora-A and Aurora-B, for Aurora kinase activity measured by Histone H3 phosphorylation at serine10 (S10-HH3), and for EGFR protein levels. (B) Upper panel: *AURORA-A* and *AURORA-B* transcript levels were assessed by real-time qRT-PCR. Shown is the relative expression normalized to the expression of *Ubiquitin*. Lower panel: Expression of EGFR analyzed by RT-PCR. None of the SCCHN cell lines express the EGFRvIII mutant. Transiently transfected NIH-3T3 cells expressing EGFRvIII (3T3-EGFRvIII) were included as a control. (C) Upper panel: CAL cells were treated with 200 nM Cetuximab for the indicated time and assessed by immunoblotting for suppression of EGFR downstream target phosphorylation. Lower panel: Treatment of FADU cells with 5 nM Pan-Aurora kinase inhibitor R763 for the indicated time. The activity of Aurora kinases was assessed by immunoblotting for S10-HH3. (D) SCCHN cell lines were treated for 24 hr with R763 at the indicated concentrations or carrier alone (0 nM). The representative histograms show the DNA content assessed by propidium iodide (PI) staining.

Given our results (Figure 3) and mRNA data showing that Aurora-A expression is an adverse prognostic factor [21], molecular targeted therapy towards Aurora kinases could be an attractive approach. We first characterized six SCCHN cell lines for the expression of EGFR, Aurora-A and Aurora-B. As expected all cell lines showed detectable levels of Aurora kinases as well as phosphorylation of the Aurora kinase substrate Serin10-phosphorylated Histone H3 (S10-HH3) [23](Figure 4A). Real-time PCR analysis revealed no clear correlation between transcript and protein level for Aurora-A or Aurora-B (Figure 4B, upper panel). We next assessed the presence of the EGFR variant III (EGFRvIII), which has been reported to contribute to tumor growth and resistance to EGFR targeting [24]. EGFRvIII was not present in any of the cell lines analyzed by RT-PCR, where NIH-3T3 cells that were engineered to ectopically express EGFRvIII were included as a control (Figure 4B, lower panel). We next analyzed the effects of the EGFR antibody cetuximab and the small molecule pan-Aurora kinase inhibitor R763 [25] on SCCHN cells. Treatment with 200 nM cetuximab resulted in reduced autophosphorylation of EGFR after 5 minutes, which subsequently resumed to normal and above normal levels consistent with a previous report [26]. In accord, the abundance of phosphorylated Akt and Erk upon cetuximab treatment was reduced [27] (Figure 4C, upper panel). We then assessed the abundance of S10-HH3 as a measure of Aurora kinase activity. The exposure to 5 nM R763 lead to a rapid and efficient decrease in S10-HH3 levels (Figure 4C, lower panel). In order to assess the Aurora kinase inhibition effects on ploidy and cell death we next treated SCCHN cell lines for a 24 hour period with R763 at various concentrations. There was a strong effect with regard to G2-M arrest and/or ploidy (p<0.05 in all cell lines, Figure 4D) and to a lesser extent to the subG1 fraction of SCCHN cells, indicating that mitosis and cytokinesis were effectively blocked. R763 treatment did however result in low apoptosis rates. In conclusion, a low nanomolar concentration of the Aurora kinase inhibitor R763 resulted in effective inhibition of Aurora kinase activity, of cytokinesis and caused polyploidy.

### Additive effects of combined Aurora kinase and EGFR targeting

**Figure 5:**
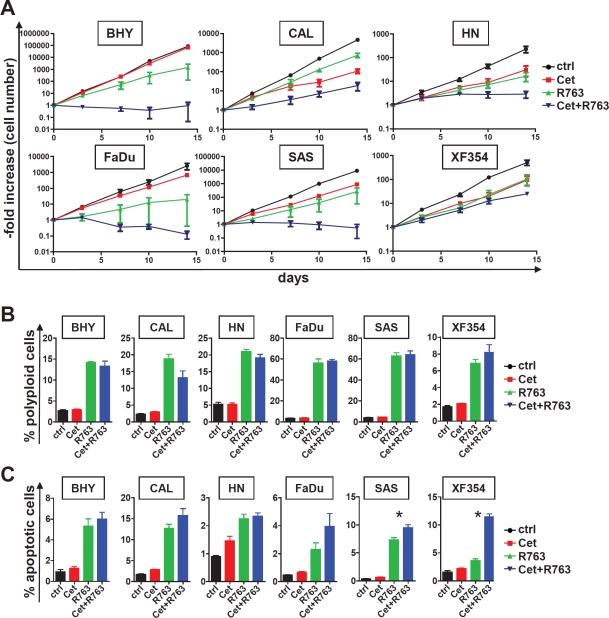
Combined exposure to EGFR antibody and Aurora kinase inhibitor results in fortified growth inhibition and apoptosis (A) SCCHN cells were treated for a total of 14 days with cetuximab (200 nM), the Aurora kinase inhibitor R763, the combination of both (Cet+ R763), or carrier only (control). The cell number was counted at the indicated times and the -fold increase in cell number calculated. Note that the increase in cell number is given in a logarythmic scale. The combination of cetuximab and Aurora kinase inhibitor resulted in a significantly reduced -fold increase after 14 day treatment period in all cell lines investigated in comparison to all other conditions (Cet alone, R763 alone, control; p<0.05). (B) The indicated SCCHN cells were cultured for a 48 hr period with the indicated conditions (cetuximab 200 nM, R7635 nM) and assessed for DNA content by PI staining. The percentage of polyploid cells with a DNA content >4n is given. (C) Analysis of the cells shown in (B) for apoptosis (Annexin V-positive, PI-negative cell fraction) by flow cytometry. The bars represent the mean ± SD of 3 independently performed experiments. Statistically significant differences are marked (* indicates p<0.05).

Given that we found Aurora-A and EGFR protein expression as adverse prognostic factor in SCCHN, targeting both is an attractive therapeutic approach. We therefore assessed whether combined targeting using R763 and cetuximab would result in increased cell cycle effects and/or apoptosis. To mimic the in vivo drug action we estimated the long term effects of EGFR and/or Aurora kinase targeting in asynchronously growing SCCHN cultures. SCCHN growth curves revealed that the addition of 200 nM cetuximab or 5 nM R763 results in a delayed growth inhibition starting at around 7 days after treatment initiation (data not shown). The effects of a combination treatment in longer term cell culture were significantly pronounced (Figure 5A). Quite surprisingly, in cell lines that showed no or very moderate growth inhibition upon cetuximab only treatment (BHY and FaDu), addition of the Aurora kinase inhibitor led to an additive growth inhibition (Figure 4A), even in cells that are characterized by very low EGFR expression (BHY, Figure 4A). Thus, the combination of Aurora kinase inhibition and EGFR targeting is highly efficient in vitro and may overcome cetuximab resistance.

To mechanistically address the additive effect SCCHN cells were incubated with 5 nM R763, which blocked kinase activity effectively (Figure 4C), 200 nM cetuximab or the combination of both drugs, and compared to untreated controls. 48 hour treatment with cetuximab showed minor efficacy with regard to cell cycle arrest and polyploidy or apoptosis induction assessed by PI staining or AnnexinV positivity. 48 hour treatment with R763 resulted in a significant increase in polyploid (Figure 5A) and apoptotic cells (Figure 5B). The combination of cetuximab and R763 did not lead to a significantly increased fraction of cells with a polyploid phenotype representing defective mitosis and cytokinesis as compared to R763 monotherapy (Figure 5A), but, importantly, in several cell lines to a significantly elevated percentage of cell death (subG1 DNA content, data not shown), and AnnexinV positive apoptotic cells (Figure 5B). Thus, combined EGFR and Aurora kinase targeting results in additive effects, potentially by sensitizing mitotic checkpoints.

### Selective Aurora-A inhibition is less effective than combined Aurora kinase inhibition

**Figure 6:**
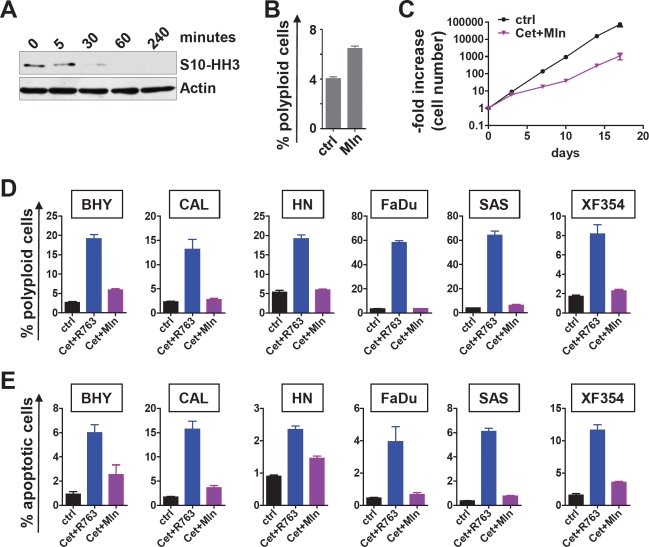
Selective Aurora-A inhibition versus pan-Aurora kinase inhibition in combination with Cetuximab (A) FADU cell were treated with 10 nM Mln for the indicated time. The effect of Aurora-A inhibition was assessed by immunoblotting for serine10-phosphorylated Histone H3 (S10-HH3). (B) Mln treatment (10 nM) for 48 hr resulted in a significant (p<0.05) but moderate increase of polyploid cells (>4n DNA content) as evaluated by PI flow cytometry. (C) Combined Aurora-A inhibiton with 10 nM Mln and EGFR inhibition with 200 nM cetuximab treatment results a significantly reduced cell number increase. (D) The indicated SCCHN cell lines were treated for 48 hr with carrier only or cetuximab plus R763 (Cet+ R763) or cetuximab plus Mln (Cet+Mln). The percentage of polyploid cells as defined by a DNA content >4n was measured by flow cytometry of PI stained cells. (E) Cells were treated as in (C). The percentage of apoptotic cells was assessed by Annexin V flow cytometry. The bars represent the mean ± SD of 3 independent experiments. The differences between Cet+ R763 versus Cet+Mln treatment are significant (p<0.05) for all cell lines tested with regard to polyploidy and with regard to apoptosis.

R763 is a pan-Aurora kinase inhibitor that inhibits Aurora-A and Aurora-B [28]. To further analyze whether Aurora-A, a prognostic factor in SCCHN [21](and this report Figure 3), or Aurora-B is the major target of R763 in SCCHN, we next directly compared R763 with the Aurora-A specific kinase inhibitor MLN8237 (Mln). Mln effectively blocked S10-HH3 phosphorylation at 10nM (Figure 6A). Mln treatment furthermore resulted in an increase of the fraction of polyploid cells (Figure 6B), and combined EGFR and Aurora-A targeting using Mln decreased the growth of SCCHN cells significantly (Figure 6C). A direct comparison of the Pan-Aurora kinase inhibitor R763 (5nM) and the Aurora-A specific kinase inhibitor Mln (10nM) at concentrations that each block S10-HH3 phosphorylation effectively revealed that the R763/cetuximab combination was much more potent in inducing polyploidy (Figure 6D) as well as apoptosis (Figure 6E) compared to cetuximab in combination with the specific Aurora-A inhibitor Mln. Thus, the superior effects of R763 are most likely mediated by its blockage of Aurora-B activity or its dual Aurora kinase inhibition.

## DISCUSSION

Other than EGFR blockage through cetuximab, none of the targeted approaches have yet shown clinically convincing results or changed the standard of care in relapsed or metastatic SCCHN. We identify the Aurora kinases as potential targets in this disease. Aurora kinases are upregulated in multiple human cancers, correlating in some cases with poor prognosis [18, 20-21, 29]. By investigating 180 patient samples of SCCHN tumors we show that both Aurora-A and EGFR are significantly overexpressed in tumor tissue. The spearman correlation coefficient showed that the expression of Aurora-A and EGFR was independent. Our findings thus establish that the joint overexpression of EGFR and Aurora-A defines a subgroup of SCCHN patients with inferior prognosis regarding disease-free and overall survival. These results prompt the analysis of combined targeted treatment strategies in this disease. We used a dual Aurora-A/Aurora-B inhibitor in combination with EGFR blockage through cetuximab and established an additive or possibly even synergistic effect on SCCHN cells in vitro. At this time it is however not clear whether Aurora-B was the main therapeutic target in our SCCHN studies or whether combined inhibition of Aurora-A and Aurora-B is beneficial. In a targeted small interfering RNA screen others identified Aurora-A as a component of an EGFR-centered network. When the Aurora kinase inhibitor PHA-680632 (PHA) was combined with EGFR inhibition, therapeutic synergism was observed in EGFR-dependent cell lines [30]. It has however to be noted that the applied concentrations of PHA most likely also inhibit Aurora-B [31]. There is further linkage between EGFR activation and Aurora-A. A study demonstrated that the nuclear EGFR can cooperate with STAT5A to target the promoter region of *AURORA-A* and enhance its expression in cancer cells [32].

A consistent finding in our in vitro study is that there is a uniform additive inhibition of cell growth when cetuximab and Aurora kinase inhibition was combined, even in cell lines that were resistant towards EGFR-directed treatment or that showed moderate growth inhibition upon single Aurora kinase targeting. Our immunohistochemical studies did not address the frequency of the EGFRvIII mutant that might be associated with resistance towards cetuximab [24]. The cell lines we used did not express EGFRvIII. At this time we cannot conclude whether EGFRvIII bearing SCCHN patients have an inferior prognosis (our retrospective cohort) or whether EGFRvIII mutant cell lines are different with regard to sensitivity towards Aurora kinase inhibition. A recent clinical trial indicated that high EGFRvIII expression levels identify SCCHN patients who are less likely to benefit from combination treatment with cetuximab and docetaxel [33]. However, our studies suggest that even inhibiting a very low level of EGFR expression might be sufficient to sensitize for Aurora kinase inhibition. This could occur by either concertedly targeting the same growth and/or survival pathways or by blocking resistance-mediating mechanisms.

The G2-M targeting approach is of particular interest since conventional chemotherapy usually targets cancer cells at the G1-S transition of the cell cycle. The cell cycle is driven by Cyclin-dependent kinases (Cdk). Of particular importance is the negative regulation of Cdk by checkpoints when defects such as DNA damage occur. Following DNA damage the transcription factor p53 is activated, which results in transcription of the Cdk inhibitor p21 and cell cycle arrest in G1, or induction of apoptosis [34]. Loss of p53 function, a frequent event in SCCHN [4, 35], therefore has the dual effect of loss of the G1-S checkpoint and loss of an important pathway leading to death [36]. On the other hand G2-M checkpoint genes are rarely if ever mutated in cancer. Therefore therapeutics targeting cancer cells at G2-M and during cytokinesis are highly interesting. Current therapeutic strategies in SCCHN use mitotic poisons such as taxanes, which act directly on spindle microtubules inducing spindle assembly checkpoint (SAC) activation, and prolonged mitotic arrest that frequently ends in cell death [37]. A second approach is to directly target mitotic checkpoint kinases such as Aurora kinases. Several of the currently available Aurora kinase inhibitors target both Aurora-A and Aurora-B. Comparing the pan Aurora kinase inhibitor R763 [25] with the Aurora-A specific inhibitor MLN our results establish Aurora-B as the potentially more powerful target in SCCHN, but cannot rule out that a combined Aurora-A and Aurora-B inhibition might be beneficial to induce mitotic failure and cell death. Importantly, there are interactions between Aurora-A and p53, where Aurora-A directly phosphorylates p53 to augment p53 protein turnover and transcriptional activity [38]. In addition, a differential effect of Aurora kinase inhibition related to function p53 has been suggested [39].

The G2-M checkpoint is a particularly interesting therapeutic target in SCCHN, where due to the high frequency of mutations in the p53 apoptotic pathway the G1-S checkpoint is often dysfunctional. Our results define a new risk group in SCCHN and provide a rationale for testing combined EGFR and Aurora kinase targeting in clinical studies.

## MATERIALS AND METHODS

### Patient selection and tissue samples

Paraffin wax-embedded tumor samples from 180 patients (mean age 54 years, range 30-70 years) with a squamous cell carcinoma of the oral cavity, oropharynx, hypopharynx and larynx were investigated. Patients had been treated by radical surgical resection between 1993 and 1997 in the Department of Head and Neck Surgery, Klinikum rechts der Isar, Technische Universität München (TUM), Munich, Germany or in the Department of Head and Neck Surgery, University of Regensburg, Regensburg, Germany.

The pT and pN categories of the tumor were determined according to the tumor-node-metastasis classification [40] and tumor grading according to the World Health Organization (WHO) classification [41]. For all tumors and patients, histopathological and clinical follow-up data were available (mean follow-up period 6.6 years, follow-up period of 0.02 to 13.6 years). Clinical and histopathological data were correlated with expression patterns of Aurora-A and EGFR. The study was approved by the Ethics Committee of the Medical Faculty of the TUM. Detailed patient characteristics and histomorphological features are shown in Table 1.

### Preparation of Tissue MicroArrays (TMA), Immunohistochemistry (IHC), and Scoring

For each of the 180 SCCHN, one paraffin block was selected. An experienced pathologist marked the viable, representative areas of tumor specimens. Core needle biopsy specimens were retrieved from the original tumor blocks by using a manual arrayer (Beecher Instruments, Sun Prairie, WI, USA) and positioned in a recipient paraffin wax array block. We aimed to obtain at least three tissue cylinders per tumor with a diameter of 0.6 mm from each biopsy specimen.

IHC was performed on deparaffinized tissue sections (2 μm), stained with antibodies against Aurora kinase A (Novocastra, Leica-Microsystems, Wetzlar, Germany) and EGFR (Santa Cruz Biotechnology, Santa Cruz, CA), visualized with peroxidase-conjugated secondary antibody (LSAB Kit, DAKO, Hamburg, Germany). The tissue sections were counterstained with Mayer hematoxylin solution. For positive controls, we used tissues with known expression of the respective antigens. For negative controls, we used irrelevant antibodies with the same immunoglobulin isotype.

According to previously published criteria cytoplasmatic and/or nuclear immunoreactivity of Aurora-A [21] and the membrane and/or cytoplasmatic staining of EGFR [42] was evaluated in three tumor areas of each case. Immunoreactivity was scored into seven groups according to the percentage and intensity of cytoplasmic, nuclear and membrane staining of the positively stained tumor cells. Specimens with > 60% of cells stained were scored as strongly positive (4+), those with 30-60% of cells stained were scored as moderately positive (3+), those with 10-20% of cells stained were scored as weakly positive (2+), those with < 10% cells stained were scored as less weakly positive (1+). Specimens with no staining were scored as negative. The intensity of staining was grouped in strong (3+), moderate (2+) and weak (1+). Intensity and percentage of staining cells were added up identifying the seven groups. All scoring analysis was done by two independent investigators. To compare high with low expression levels, a median split analysis was applied. EGFR≥5 and Aurora-A≥5 were specified as high expression.

### Cell culture, transfection and plasmids

All cell lines were obtained from ATCC-LGC (Wesel, Germany) or DSMZ (Braunschweig, Germany). SCCHN cells were cultured in DMEM (Invitrogen, Darmstadt, Germany) supplemented with 10% heat inactivated fetal bovine serum (FBS, PAA, Cölbe, Germany), 1% glutamine, 1% penicillin-streptomycin and 1% non-essential amino acids (all from Invitrogen, Darmstadt, Germany). NIH-3T3 cells were cultured in DMEM supplemented with 10% heat inactivated bovine serum and 1% penicillin-streptomycin. NIH-3T3 cells were transfected with pLERN-EGFRvIII (kind gift of Frank Furnari, La Jolla, CA) with Lipofectamine 2000 according to the manufacturer's instructions (Invitrogen, Darmstadt, Germany) and selected with G418 (1000 μg/ml, Sigma, Munich, Germany). To measure proliferation, SCCHN cells were split, reseeded (5×10^5^in 25cm^2^ flasks), and counted at the indicated time points. Cells were then replated at the initial density. The -fold increase in cell number was calculated, all given results are based on triplicate experiments. R763 [25] was kindly provided by EMD-Serono (Rockland, MA). MLN8237 was purchased from Selleck (Houston, TX).

### Flow cytometry and apoptosis assays

To assess apoptosis, 5×10^5^ cells were stained with FITC-labeled Annexin V (BD Pharmingen, Heidelberg, Germany) and counterstained with propidium iodide (PI, Sigma, Munich, Germany). Following incubation cells were washed, resuspended in PBS, and analyzed by flow cytometry. The fraction of Annexin V–positive (FL2 channel) PI-negative (FL3 channel) cells was reported as apoptotic. For analysis of cell cycle distribution, cells were fixed with 70% ethanol and stained with PI. Flow cytometric analysis of DNA content was performed using PI in the FL2 channel in linear mode. Cells with less than diploid DNA content were considered dead (sub-G1), cells with more than 2N DNA content were considered polyploid.

### RNA preparation and analyses

For reverse transcriptase quantitative PCR (qRT-PCR), RNA was prepared from cultured cells using the RNeasy kit (Qiagen, Hilden, Germany). cDNA was prepared from 2 μg RNA using the SuperScript II reverse transcriptase cDNA synthesis kit (Invitrogen, Darmstadt, Germany). qRT-PCR was performed on an ABI Prism 7900 cycler (Applied Biosystems, Darmstadt, Germany) with the Platinum SYBR Green qPCR SuperMIX-UDG kit (Invitrogen, Darmstadt, Germany). Data were analyzed by using the ΔCt method, where *Ubiquitin* served as an internal control, with one sample set as 1. RT-PCR was performed to validate the expression of the EGFRvIII mutant in NIH-3T3 cells. Primer sequences can be obtained from the authors upon request.

### Immunoblotting

Protein extracts (50 μg per lane) were electrophoretically separated on SDS-PAGE gels, transferred to membranes (Protran, Schleicher&Schuell, Dassel, Germany) and blotted with specific antibodies as described earlier [43] (Actin, Aurora-A, Aurora-B: all from Sigma, Munich, Germany; S10-HH3: Millipore, Schwalbach, Germany; EGFR: Santa Cruz, Heidelberg, Germany; pEGFR: Invitrogen, Darmstadt, Germany, pAKT, pERK: both from New England Biolabs, Frankfurt, Germany).

### Statistical analysis

Statistical analyses were performed using the statistical functions (t-test if not otherwise indicated) of GraphPad Prism (GraphPad Software, La Jolla, CA). For quantitative variables, means and standard deviations are given, for categorical data absolute and relative frequencies. The bars shown represent the mean ± standard deviation (SD) or standard error of the mean (SEM). Spearman rank correlation coefficient (r) was correlated to assess the relationship between Aurora-A and EGFR expression. Also for box plots showing medians, quantiles and ranges as well as Kaplan-Meier survival analyses the Statistical Package for Social Sciences (SPSS 17.0 Package Facility, SPSS Inc, IL, USA) was used. Survival curves were compared with the log-Rank test. Any *p* values given are two-sided and subject to a local significance level of 0.05.
